# Case Report: Toxic epidermal necrolysis associated with sintilimab in a patient with relapsed thymic carcinoma

**DOI:** 10.3389/fonc.2022.1065137

**Published:** 2022-12-22

**Authors:** Huayu Yang, Qing Ma, Ying Sun, Kan Zhang, Yunli Xing, Hongwei Li

**Affiliations:** ^1^ Department of Geriatrics, Beijing Friendship Hospital, Capital Medical University, Beijing, China; ^2^ Department of Cardiology, Beijing Friendship Hospital, Capital Medical University, Beijing, China

**Keywords:** thymic carcinoma, immunotherapy, sintilimab, immune-related adverse events, toxic epidermal necrolysis

## Abstract

Immune checkpoint inhibitors (ICIs) such as anti-programmed death 1 (PD-1) receptor monoclonal antibody has been shown to be effective in patients with relapsed thymic carcinoma. However, immune-related adverse events (irAE) are increasingly recognized. There is a paucity of clinical data, especially in elderly patients. A patient in his late 80s with a history of thymic carcinoma was treated with sintilimab, an anti-PD1 antibody. After one week of administration, the patient developed diffuse rash. After two cycles of sintilimab, there was rapid progression of the rash with gradual development of blisters and skin detachment. Sintilimab was immediately discontinued, and skin biopsy was performed. The histopathological findings were consistent with the diagnosis of toxic epidermal necrolysis (TEN), which was considered as an irAE. Intravenous methylprednisolone was initially administered, followed by oral prednisone. The patient showed dramatic improvement within 72 hours of initiation of treatment. Unfortunately, the patient died of severe pneumonia three months later. We report a case of TEN, a rare toxicity induced by anti-PD-1 sintilimab in an elderly patient with thymic carcinoma. Since TEN is a life-threatening condition, early recognition and management of this complication is a key imperative.

## Case report

An 82-year-old man was admitted to the hospital due to hoarseness of voice. Six years ago, he was diagnosed with thymic squamous cell carcinoma with pericardial metastases. He was treated with a wide local excision, but did not receive chemotherapy or radiation therapy. The disease stage was IVA according to the Masaoka⁃Koga staging criteria. One year later, the patient was lost to follow-up. He had outpatient medications for mild anemia and leukopenia (leukocyte count: 2.20×10^9^ cells/L; hemoglobin 100 g/L).

Physical examination revealed left supraclavicular lymph node enlargement. Chest CT showed soft-tissue enlargement of the anterior mediastinal ascending aorta. Biopsy of left supraclavicular lymph node confirmed squamous cell carcinoma of thymic origin. The cause of hoarseness was considered to be tumor recurrence involving the recurrent laryngeal nerve. The oncologist recommended radiotherapy for the patient, however, the family refused. He finally opted for targeted therapy with anti-PD1 receptor monoclonal antibody.

**Figure 1:**
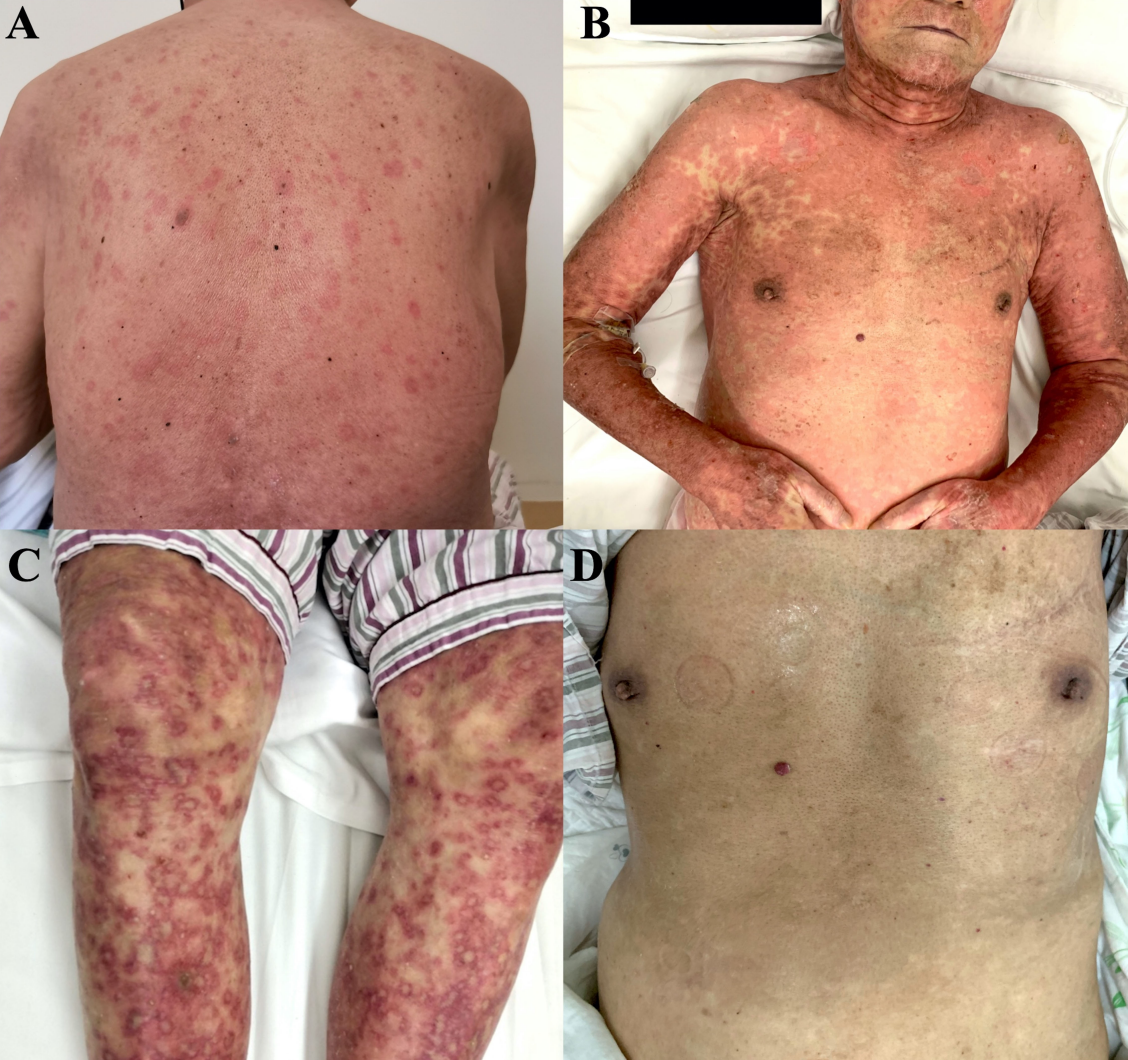
Photographs of skin lesion induced by sintilimab in an elderly patient with relapsed thymic carcinoma. **(A)** Rashes after first cycle of treatment. **(B, C)** Rashes with blisters and skin detachment after second cycle of treatment. **(D)** Skin after recovery from severe toxic epidermal necrolysis.

**Figure 2:**
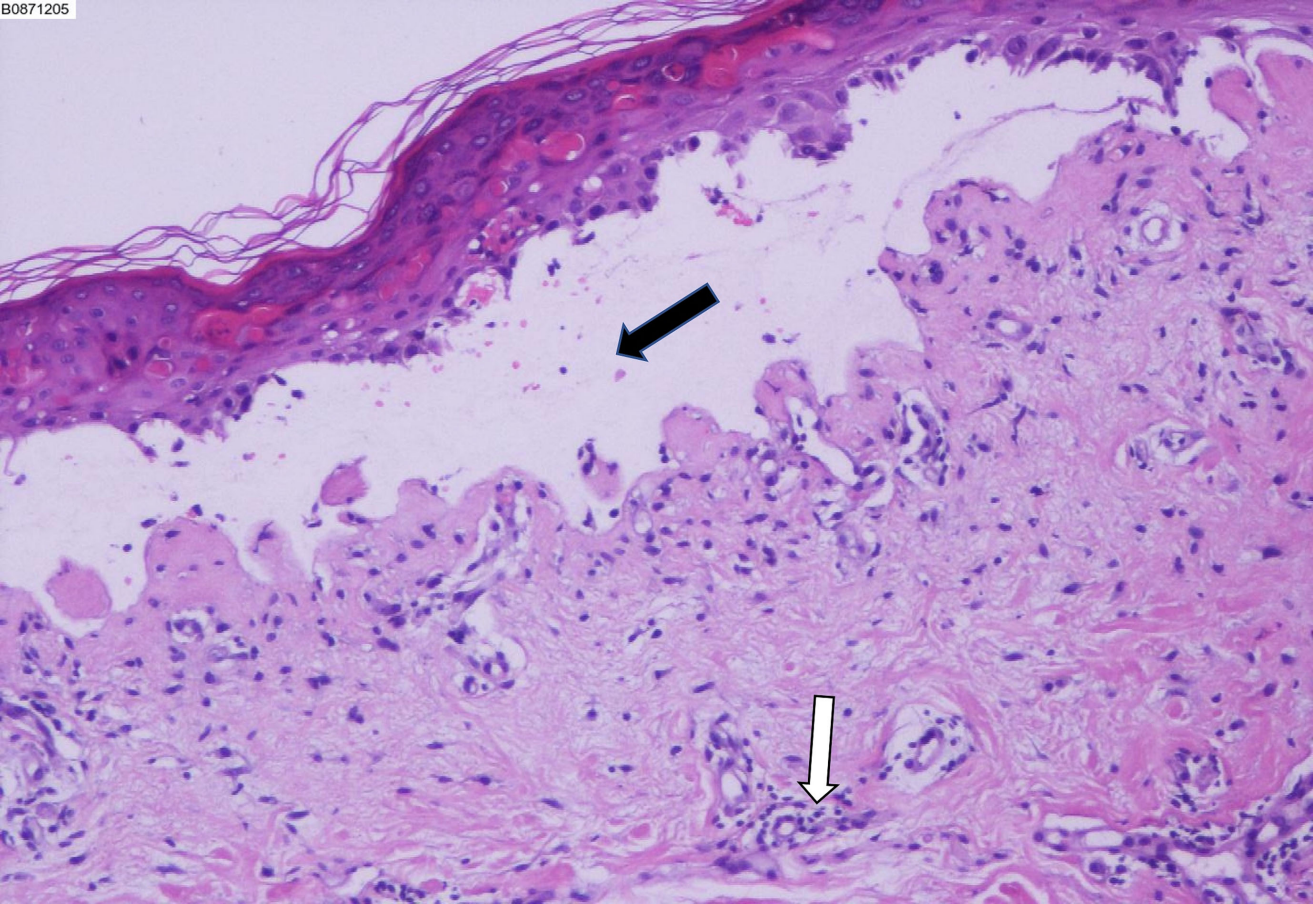
Biopsy of the skin lesion showing detachment of the epidermis and mucous membrane, which confirmed toxic epidermal necrolysis. Black arrow indicates subepidermal blisters. White arrow indicates perivascular lymphocytes accumulation.

**Figure 3:**
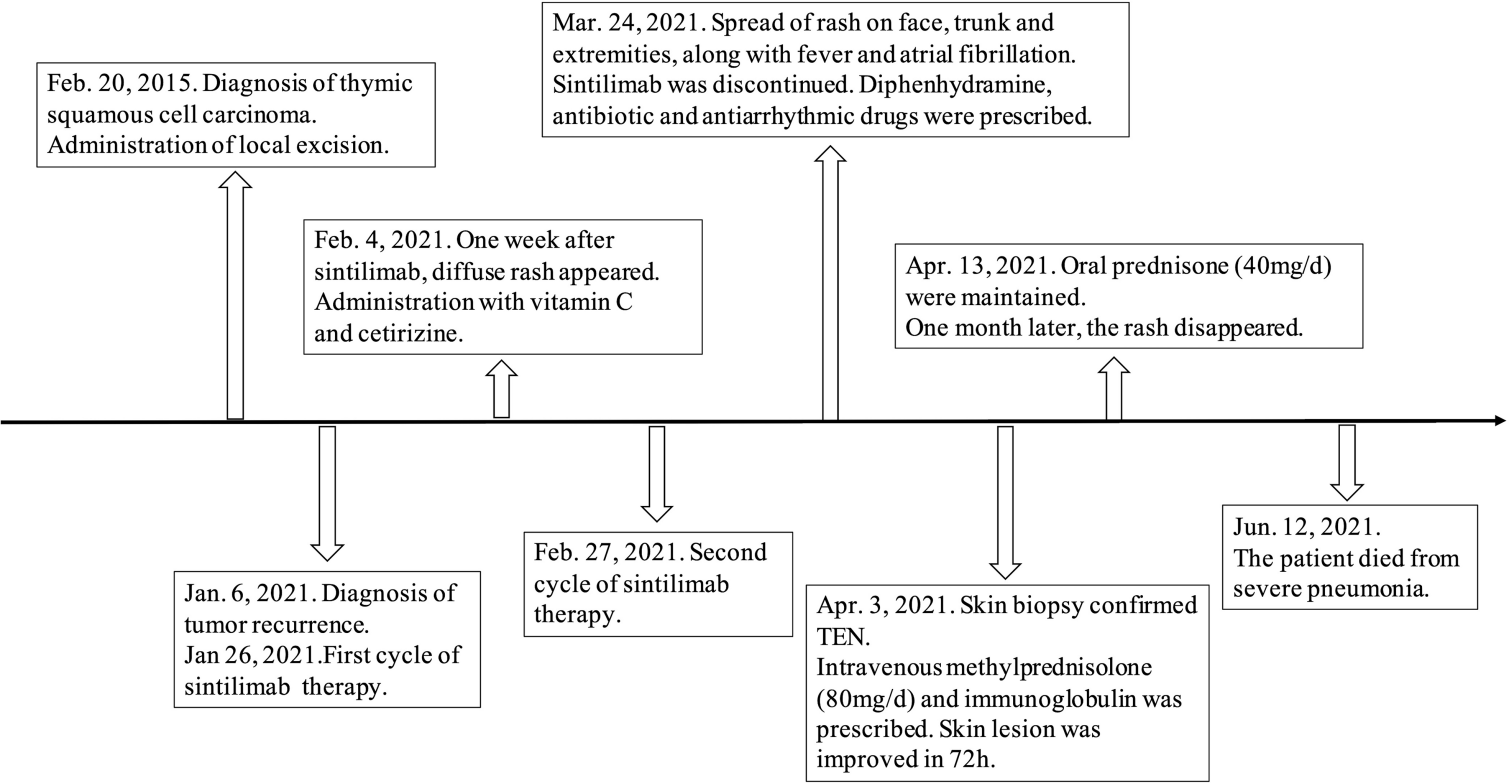
Timeline of diagnosis and major interventions. Body weight of this patient was 55 kg.

He was prescribed sintilimab every four weeks. One week after sintilimab administration, he developed diffuse rash, which improved with vitamin C and cetirizine. After the second cycle, there was recurrence of rash on the face, trunk, and the extremities, which aggravated with formation of blisters and skin detachment (**Figures 1A-C**). At this time, the patient admitted that he had experienced severe urticaria five months ago, which improved with cetirizine and ebastine. Subsequently, he presented atrial fibrillation and fever in the absence of overt signs of infection or other predisposing factors. His laboratory parameters were as follows: leukocyte count 0.22×10^9^ cells/L (normal range, 3.5–9.5×10^9^ cells/L) with 0.01 neutrophils (normal range, 1.8–6.3×10^9^ cells/L); hemoglobin 66 g/L (normal range, 130–175 g/L), platelet count 17×10^9^ cells/L (normal range, 125–350 cells/L). The patient underwent biopsy of skin lesion; pathological staining showed detachment of the epidermis and mucous membrane, which was confirmed as toxic epidermal necrolysis (TEN, **Figure 2**). The severity-of-illness score for TEN (SCORTEN) (1) was 7. Sintilimab was permanently discontinued. Intravenous methylprednisolone 1–1.5 mg/kg/day was prescribed and continued for one week. Immunoglobulin was also administered as synergistic treatment. Observable improvement of skin lesion was evident within 72h. Oral prednisone were maintained for sequential therapy and the dosage was gradually reduced. Broad-spectrum antibiotics were administered to prevent infection. One month later, the rash disappeared (**Figure 1D**), but the pneumonia worsened. Sputum culture showed Bacillus canalis capsulatus and Aspergillus flavus stenotrophomonas maltophilia. Three months later, the patient died from severe pneumonia. Timeline of diagnosis and major interventions is shown in **Figure 3**.

## Discussion

TEN is a life-threatening disease (reported mortality rate: 14.8%) histopathologically characterized by detachment of the epidermis and mucous membrane. It is a drug-induced disease, most frequently caused by antibiotics, allopurinol, non-steroidal anti-inflammatory drugs, and antiepileptic drugs. However, TEN induced by PD-1-targeted therapy has rarely been reported. Different from traditional cytotoxic chemotherapy, the typical PD-1 immune-mediated adverse events include colitis, hepatitis, thyroiditis, pituitary inflammation, pneumonia, pericarditis, hypothyroidism, nephritis, fatigue, and rash (2). A retrospective study reported that PD-1 immune-mediated TEN often appeared several days to weeks after treatment, with a median time of 4 weeks. The rash is often non-specific, and mimics erythema, measles-like rash, or radioactive dermatitis (3). Early identification is the key to the timely management of this condition.

Until now, only few cases have been reported that sintilimab induced TEN in patients with malignancies (4–6). The therapy and time periods of adverse reactions were different. Two patients were performed on monotherapy of sintilimab. One case of metastatic gallbladder carcinoma patient showed TEN appearing after receiving first dose of sintilimab (4), whereas our present case developed TEN after two cycles of administration. In other two cases, sintilimab was administrated in combination with chemotherapy, and both patients suffered TEN when fourth chemoimmunotherapy was finished (5, 6). All the patients were quickly prescribed with glucocorticoid and immunoglobulin when TEN was diagnosed. Except for the oldest patient who died of infection three month later mentioned in this paper, other patients were discharged after improvement.

The specific mechanism about ICIs-related TEN still remains unclear. In the traditional theory, the trigger of TEN may relate to T cells-mediated cytotoxic reaction. However, the over-activation of immune system gradually gains importance. Histopathological results show the keratinocytes present molecular and morphological characteristics of apoptosis in the early stage of TEN. The following characteristic is full-layer epidermal necrosis. These pathological findings exhibit similar to graft-versus host reaction (GVHR) (7). Thus, some experts speculate that the administration of ICIs may induce graft‐versus‐host‐disease (GVHD) reaction that results in widespread autoimmunity in most patients (8). In a retrospective analysis, R. Kleef. et al. pointed out that a low-dose-combination of ICIs was safer than that of protocols without compromising efficacy (9). More researches are needed to confirm these theories in the future.

Sintilimab, a monoclonal antibody against PD-1, has been shown to be effective in the treatment of relapsed Hodgkin lymphoma, non-small cell lung cancer, digestive system cancers, urothelial carcinoma, and head and neck squamous cell carcinoma (10). Previous studies have revealed the involvement of programmed cell death protein 1/programmed death-ligand 1 (PD-1/PD-L1) pathway in the progression of thymic epithelial tumors, and it was a predictor of response. This provided a strong rationale for use of ICIs for treatment of thymic epithelial tumors (11). However, a higher incidence of immune-related adverse events has been reported in patients with thymic epithelial tumors compared to patients with other cancers (12). The thymus is an immune organ. Patients with thymic carcinoma frequently have immune dysfunction, which makes them more likely to develop severe immune responses. Due to the low incidence of thymic carcinoma, there is a paucity of clinical data on the application of ICIs in these patients. At the moment, ICIs are not the standard of care for patients with thymic epithelial tumors and larger trials are required to provide more robust evidence. Our patient was in a hypersensitive state while receiving ICIs treatment, which may have contributed as the trigger for TEN.

Checkpoint antibody inhibitors are a novel class of cancer inhibitors which act *via* modulation of immune cell-tumor cell interaction. Despite the huge success, their efficacy is limited to specific types of cancers. Due caution should be exercised because of the risk of immune-related adverse events, especially in patient with allergic history.

## Conclusion

ICIs are emerging as a powerful weapon against various cancers. However, due cognizance should be taken of drug toxicities and immune-related adverse events. In this paper, we describe an elderly patient who experienced TEN after sintilimab targeted therapy. After treatment with corticosteroids, the patient’s rash was effectively resolved, but he died of severe pneumonia during steroid administration. It is necessary to fully evaluate the benefits of ICIs, especially in patients with thymic carcinoma and those in a hypersensitive state. Closely monitoring during the treatment is crucial to identify adverse reactions in a timely manner.

## Data availability statement

The original contributions presented in the study are included in the article/supplementary material. Further inquiries can be directed to the corresponding authors.

## Ethics statement

The studies involving human participants were reviewed and approved by Ethics Committee of Beijing Friendship Hospital, Capital Medical University, and the approval number is 2022-P2-168-01. The patients/participants provided their written informed consent to participate in this study. Written informed consent was obtained from the individual(s) for the publication of any potentially identifiable images or data included in this article.

## Author contributions

HY, KZ, YS and YX treated the case. HY wrote the manuscript. QM was responsible for the revision of the paper. YX and HL were in charge of final approval of the manuscript. YX and HL contributed equally to the manuscript. All authors contributed to the article and approved the submitted version.
